# A Biocompatible Colorimetric Triphenylamine- Dicyanovinyl Conjugated Fluorescent Probe for Selective and Sensitive Detection of Cyanide Ion in Aqueous Media and Living Cells

**DOI:** 10.3390/s17020405

**Published:** 2017-02-19

**Authors:** Zi-Hua Zheng, Zhi-Ke Li, Lin-Jiang Song, Qi-Wei Wang, Qing-Fei Huang, Li Yang

**Affiliations:** 1 Chengdu Institute of Organic Chemistry, Chinese Academy of Sciences, Chengdu 610041, China; Zhengzihua14@mails.ucas.ac.cn (Z.-H.Z.); wqw@cioc.ac.cn (Q.-W.W.); 2 University of Chinese Academy of Sciences, Beijing 100049, China; 3 Cancer Center, West China Hospital, Sichuan University and Collaborative Innovation Center, Chengdu 610041, China; 18280127765@163.com (Z.-K.L.); linjsong_scu@163.com (L.-J.S.)

**Keywords:** biocompatible, cell imaging, colorimetric assay, dicyanovinyl, cyanide anion

## Abstract

A colorimetric and turn-on fluorescent probe **1** bearing triphenylamine-thiophene and dicyanovinyl groups has been synthesized and used to detect cyanide anion via a nucleophilic addition reaction. Probe **1** exhibited prominent selectivity and sensitivity towards CN^−^ in aqueous media, even in the presence of other anions such as S^2−^, HS^−^, SO_3_^2−^, S_2_O_3_^2−^, S_2_O_8_^2−^, I^−^, Br^−^, Cl^−^, F^−^, NO_2_^−^, N_3_^−^, SO_4_^2−^, SCN^−^, HCO_3_^−^, CO_3_^2−^ and AcO^−^. Moreover, a low detection limit (LOD, 51 nM) was observed. In addition, good cell membrane permeability and low cytotoxicity to HeLa cells were also observed, suggesting its promising potential in bio-imaging.

## 1. Introduction

Anions play an important role in a wide range of chemical and biological processes. Given this, anion recognition has been well studied in recent years [1]. Among various anions, cyanide anion (CN^−^) is a well-known anion that is toxic to living organisms [2]. As advocated by the World Health Organization (WHO), the maximum concentration of cyanide anion in drinking water should be 1.9 µM [3]. However, CN^−^ is widely released from industry waste, certain natural plants, cigarette smoke, etc. [4], and can thus be a serious threat to people’s health and the environment. Hence, developing effective quantitative determination methods for CN^−^ is urgent and essential.

In order to quickly, easily and accurately detect cyanide anion, various analysis methods were developed, including titrimetric [5], electrochemical decices [6,7,8], voltammetric [9], fluorometric [10,11,12,13,14,15,16,17] and so on. Compared with other methods, fluorometric and colorimetric responses based on chemical reactions have attracted much attention due to their simple operation, high selectivity, high sensitivity and low cost [18,19,20,21,22]. The sensing mechanisms of fluorometric and colorimetric chemosenors for detecting CN^−^ have been reported over the past few years, including hydrogen bonding reactions [23,24,25,26,27], forming cyanide anion complexes [28,29], electron transfer reactions [30,31,32] and nucleophilic addition reactions [33,34,35,36,37,38,39,40]. Among them, the nucleophilic addition reactions generally have high selectivity for detecting CN^−^. 

Triphenylamine-based thiophene dicyanovinyl compounds, which contain an electron-donating triphenylamine moiety and a strong electron-withdrawing dicyanovinyl group, were reported as red-emitting fluorophores with large Stokes shifts [41]. In recent years, some fluorescent probes with dicyanovinyl moieties for detecting cyanide anions have been reported [42,43,44,45,46,47]. Consequently, we hypothesized that triphenylamine-based thiophene dicyanovinyl compounds could be applied to selectively detect cyanide anion. 

**Scheme 1:**
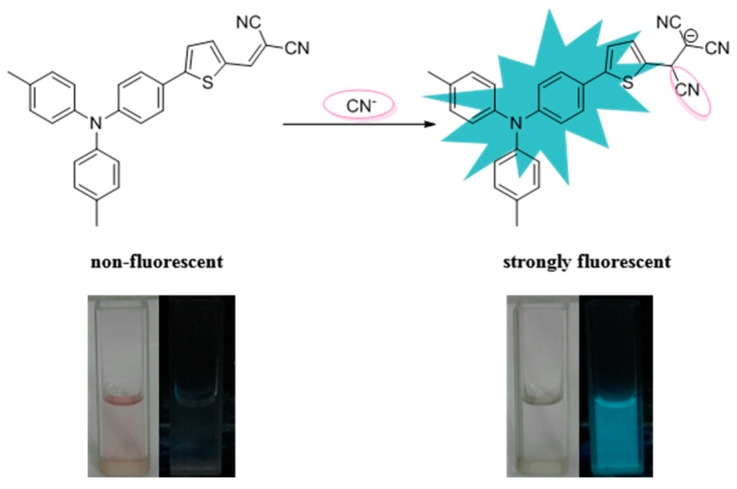
The sensing mechanism of probe **1**.

With this hypothesis in mind, probe **1** was synthesized and exhibited prominent selectivity and sensitivity towards cyanide anion with significant turn-on fluorescent response in PBS/DMSO (4/6, *v*/*v*, pH = 7.4) solution (Scheme 1). Herein, we report a dual colorimetric and fluoresecent probe to selectively and sensitively detect cyanide anion in aqueous media. Moreover, low cytotoxicity and good cell membrane permeability in HeLa cells were also observed, suggesting its promising potential in bio-imaging.

## 2. Results and Discussion

### 2.1. Synthesis of Probe **1**

**Scheme 2:**
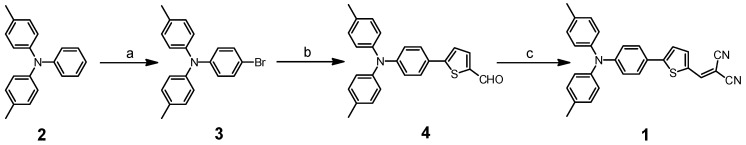
The synthesis of probe **1**; *Reagents and Conditions*: (**a**) NBS, DMF, r.t., 99% yield; (**b**) (5-formylthiophen-2-yl) boronic acid, Pd(PPh_3_)_4_, dioxane, reflux, 3.5 h, 67% yield; (**c**) malononitrile, piperidine, reflux, 3 h, 70% yield.

As shown in Scheme 2, probe **1** was synthesized via a 3-step synthesis route according to a literature method [41]. The structure of probe **1** was fully characterized by NMR and ESI-MS spectroscopy. Detailed synthetic process and structure characterization are given in the Experimental section and in the Electronic Supplementary Information (ESI).

### 2.2. Selectivity over Other Anions

**Figure 1:**
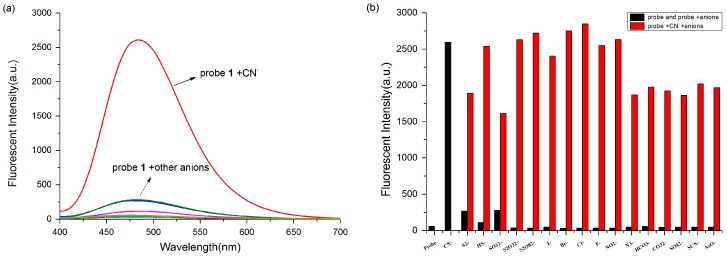
(**a**) Fluorescent responses of probe **1** (5 μM) towards various anions (50 μM) in PBS/DMSO (4/6, pH = 7.4) solution. λ_ex_ = 370 nm, Slits: 2.5 nm/5 nm; (**b**) Competing responses of probe **1** (5 μM) at 480 nm towards various analytes (50 μM) in PBS/DMSO (4/6, pH = 7.4) solution. Black bar, probe and probe + anions; red bar, probe + CN^−^ + anions. λ_ex_ = 370 nm, Slits: 2.5 nm/5 nm.

After the identification of the optimal fluorescence measurement conditions, a series of relevant and interfering anions were investigated to study the selectivity. Before the addition of various anions, the light-pink probe solution is non-fluorescent upon excited at 370 nm. The addition of 10 equivalents of 16 representative anions (S^2−^, HS^−^, SO_3_^2−^, S_2_O_3_^2−^, S_2_O_8_^2−^, I^−^, Br^−^, Cl^−^, F^−^, NO_2_^−^, N_3_^−^, SO_4_^2−^, SCN^−^, HCO_3_^−^, CO_3_^2−^ and AcO^−^) did not cause significant fluorescence changes (Figure 1a), whereas the color of the probe **1** solution faded in the presence of CN^−^ (10 equiv.) and the fluorescence intensity enhancement at 480 nm was remarkable. Based on the good selectivity of probe **1** toward CN^−^, competition experiments were further explored. As shown in Figure 1b, the fluorescence intensity of probe + CN^−^ remained at a high level by the addition of other interfering anions (10 equiv.), suggesting that the response of probe **1** toward CN^−^ has good anti-interference ability.

### 2.3. Colorimetric and Fluorescent Detection

**Figure 2:**
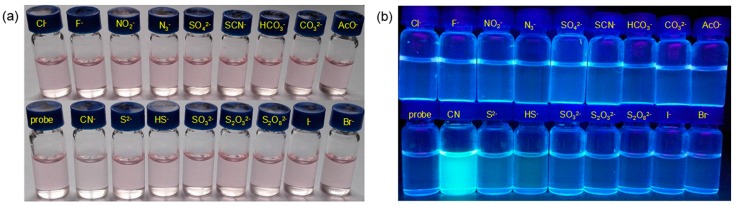
Fluorescence images of probe **1** (5 μM) upon addition of various anions (50 μM) in PBS/DMSO (4/6, pH = 7.4) solution under visible light (**a**); and 365 nm UV lamp (**b**).

The UV-vis spectra changes of probe **1** (5 μM) upon addition of CN^−^ (50 μM) in PBS/DMSO (4/6, pH = 7.4) solution can be clearly observed (Figure S1) and the absorption at 505 nm decreased. The free probe **1** exhibits absorption at 505 nm with a pink color because of ICT process in the large π-conjugation. Subsequently, a colorimetric assay was performed. As shown in Figure 2a, among tested analytes, only cyanide anion resulted in an obvious color change from light pink to colourless. Moreover, a bright blue light was achieved in the solution of probe **1** with CN^−^ under the irradiation of a UV-lamp at 365 nm, which is visible by the naked eye (Figure 2b).

### 2.4. Spectral Titration of Probe **1**

**Figure 3:**
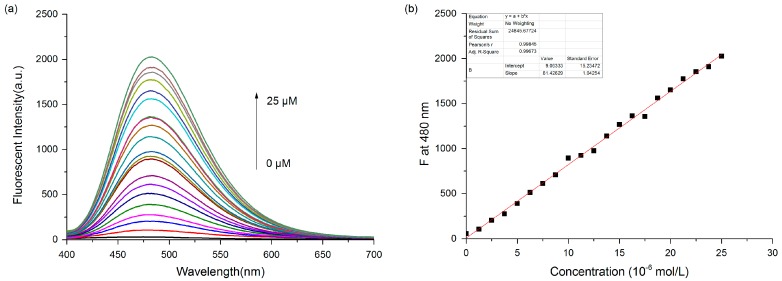
(**a**) Fluorescence spectra of probe **1** (5 μM) in the presence of various concentration of CN^−^ (0–25 μM) in PBS/DMSO (4/6, pH = 7.4) solution; (**b**) Linear relation between the fluorescent intensity at 480 nm and the concentration of CN^−^ in the range of 0–25 μM. λ_ex_ = 370 nm, Slits: 2.5 nm/5 nm.

The fluorescence titration experiment was performed by adding different concentrations of cyanide anion ranging from 0 to 75 μM to a 5 μM solution of probe **1** (Figure S3). Saturation was observed when the concentration of cyanide anion was raised to 62.5 μM. As shown in Figure 3a, a concentration-dependent fluorescence enhancement was observed when CN^−^ from 0 to 25 μM was added at 480 nm. A good linear relationship between the fluorescence intensity and the concentration of CN^−^ could be obtained in the range of 0 to 25 μM (R^2^ = 0.99698).

To calculate the limit of detection (LOD), the standard deviation of the blank measurements was determined by measuring the fluorescent intensity of probe **1** 10 times [48,49,50]. Based on the equation (LOD = 3σ/m) and the fluorescence titration, the LOD was calculated to be 51 nM, which is much lower than the maximum level of cyanide anion (1.9 µM) in drinking water permitted by the WHO, suggesting its promising potential for the detection of low levels of cyanide anion in water samples.

### 2.5. The Sensing Mechanism

**Figure 4:**
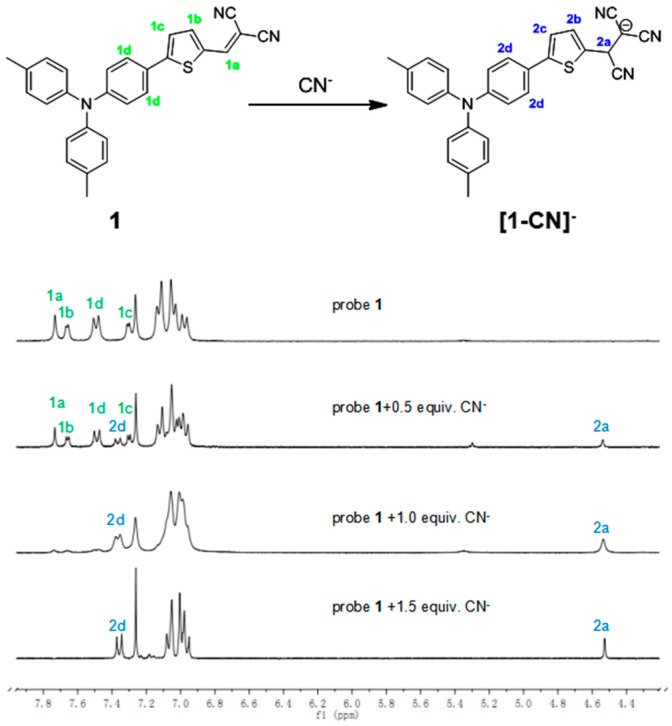
^1^H-NMR spectral changes of probe **1** (20 mM) upon addition of CN^−^ (as tetrabutylammonium salts) in CDCl_3_.

As proposed in the literature [51], the sensing mechanism of probe **1** based on a nucleophilic addition reaction has been confirmed by ^1^H-NMR spectroscopy (Figure 4). Thus, to a 20 mM solution of probe **1** in CDCl_3_ cyanide anion (0.5 equiv.) was added at room temperature and a new proton signal appeared at 4.5 ppm (H^2a^). When the amount of CN^−^ was raised to 1.0 equiv., some significant proton signal changes were observed. For example, the vinyl proton signal (H^1a^, 7.73 ppm) disappeared gradually and a new proton signal at 4.5 ppm (H^2a^) was observed. The two proton signals on the thiophene ring (H^1b^, 7.66 ppm; H^1c^, 7.30 ppm) disappeared and were observed around 7.00 ppm. In addition, the two proton signals (H^1d^) adjacent to the thiophene ring shifted from 7.49 ppm to 7.36 ppm. These important proton signal changes conformed with the reference. While 1.5 equiv. CN^−^ was added, the reaction accomplished completely. In addition, the **[1 − CN]**^−^ adduct was characterized by mass spectrometry analysis, and the peak at *m*/*z* 457.1471 (calc. 457.1492) corresponding to **[1 − CN]**^−^ was clearly observed (Figure S10).

### 2.6. Cellular Imaging

**Figure 5:**
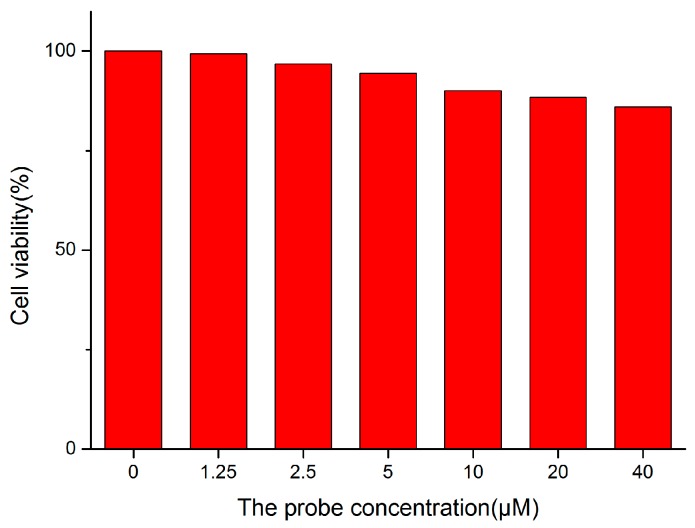
HeLa cell viability values (%) estimated by MTT assay at different concentrations of probe **1** for 24 h.

To explore its potential in bio-imaging, the cytotoxicity of probe **1** was investigated by a standard MTT assay. As shown in Figure 5, HeLa cell viability was slightly reduced when the probe concentration was raised up to 40 µM, indicating acceptable cytotoxicity of probe **1**.

**Figure 6:**
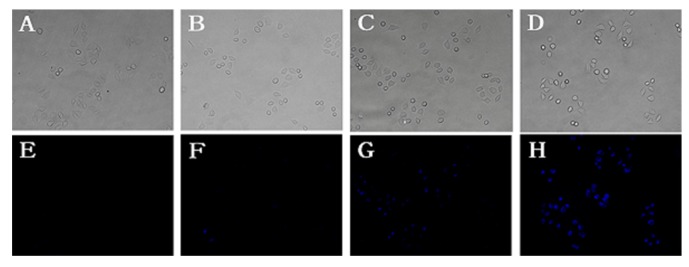
Images of living HeLa cells: HeLa cells incubated with probe **1** (10 μM) for 1 h and then with different concentration of CN^−^ ((**A**,**E**) 0 μM; (**B**,**F**) 10 μM; (**C**,**G**) 80 μM; (**D**,**H**) 160 μM) for 15 min. (**A**–**D**) is Bright field; (**E**–**H**) is under fluorescence (λ_ex_ = 385 nm, λ_em_ = 405–440 nm) blue channel (425 nm–520 nm), scale bar: 20 μm.

**Figure 7:**
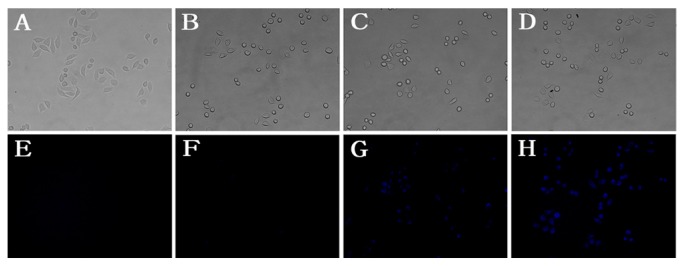
Images of living HeLa cells: HeLa cells incubated with different concentration of probe **1** ((**A**,**E**) 0 μM; (**B**,**F**) 2.5 μM; (**C**,**G**) 5 μM; (**D**,**H**) 10 μM) for 1 h and then all cells were incubated with 80 µM CN^−^ for 15 min. (**A**–**D**) is Bright field; (**E**–**H**) is under fluorescence (λ_ex_ = 385 nm, λ_em_ = 405–440 nm), scale bar: 20 μm.

Probe **1** was next applied to detect cyanide anion in living HeLa cells. As shown in Figure 6, HeLa cells were incubated with probe **1** (10 µM) for 1 h and then with different concentrations of CN^−^ (A, E: 0 µM; B, F: 10 µM; C, G: 80 µM; D, H: 160 µM) for 15 min. Under the fluorescent microscope, remarkable intracellular blue fluorescence was observed (Figure 6G–H). Moreover, the fluorescence intensity showed a concentration-dependent manner. As shown in Figure 7, HeLa cells were incubated with different concentrations of probe (0 µM; 2.5 µM; 5.0 µM; 10 µM) for 1 h and then with CN^−^ (80 µM) for 15 min. The fluorescence intensity also showed a concentration-dependent manner. In summary, probe **1** showed the potential to detect CN^−^ in vitro cellular systems in a concentration-dependent manner for both probe and CN^−^.

## 3. Experimental Section

### 3.1. Materials and Instruments

All reagents were of analytical grade and used without further purification. The absorption and fluorescence spectra were measured on a UV-2450 (Shimadzu, Kyoto, Japan) instrument and a F-7000 (Hitachi, Tokyo, Japan) fluorescence spectrometer. ^1^H-NMR (300 MHz) and ^13^C-NMR (75 MHz) spectra were obtained on a Bruker-300 MHz spectrometer (Bruker, Fällanden, Switzerland) using tetramethylsilane (TMS) as an internal standard. The following abbreviations were used to denote the signal multiplicities: s for singlet, d for doublet, t for triplet, q for quartet, m for multiplet, br for broad. High-resolution mass spectra were obtained on a micrOTOF-Q mass spectrometer (Bruker, Bremen, Germany). Fluorescence cell imaging was performed with a fluorescence microscope (Olympus, Tokyo, Japan) with a 40× objective lens.

### 3.2. Synthesis of Probe **1**

#### 3.2.1. Synthesis of 4-bromo-N,N-di-p-tolylaniline (**3**)

4-Methyl-*N*-phenyl-*N*-(*p*-tolyl)aniline (1.00 g, 3.66 mmol) and NBS (0.68 g, 3.84 mmol) were dissolved in CHCl_3_ (20 mL). The mixture was stirred for 1.5 h at room temperature. The mixture was poured into water (50 mL) and then extracted with dichloromethane. The organic layer was washed with plenty of brine and dried over anhydrous sodium sulfate. The solvent was evaporated by a rotary evaporator. A white solid (1.28 g) was obtained. Yield: 99%.^1^H-NMR (CDCl_3_): δ 7.26 (d, *J* = 9.0 Hz, 1H), 7.06 (d, *J* = 8.2 Hz, 2H), 6.96 (d, *J* = 9.0 Hz, 2H), 6.87 (d, *J* = 6.0 Hz, 1H), 2.30 (s, 3H).

#### 3.2.2. Synthesis of 5-(4-(di-p-tolylamino)phenyl)thiophene-2-carbaldehyde (**4**) 

Dioxane (50 mL) was placed in a 3-necked round bottom flask and was purged with nitrogen for 30 min. Under a nitrogen atmosphere, compound **3** (1.00 g, 2.84 mmol), (5-formylthiophen-2-yl)boronic acid (1.35 g, 3.40 mmol), K_2_CO_3_ (2.00 g, 5.68 mmol) and Pd(PPh_3_)_4_ (164 mg, 0.14 mmol) were added. The mixture was violently refluxed for 3.5 h. After cooling to room temperature, the solvent was evaporated by a rotary evaporator. The residue was purified by column chromatography (silica gel, ethyl acetate/petroleum ether = 1:20) to give the compound **4**. The product was obtained as a yellow solid (0.73 g). Yield: 67%. ^1^H-NMR (DMSO-*d*_6_): δ 9.84 (s, 1H), 7.93 (d, *J* = 4.0 Hz, 1H), 7.62 (d, *J* = 7.6 Hz, 2H), 7.56 (d, *J* = 4.0 Hz, 1H), 7.15 (d, *J* = 8.2 Hz, 4H), 6.98 (d, *J* = 8.3 Hz, 4H), 6.86 (d, *J* = 8.8 Hz, 2H), 2.27 (s, 6H); ^13^C-NMR (DMSO-*d*_6_): δ 183.6, 153.2, 148.9, 143.9, 140.6, 139.5, 133.5, 130.3, 127.3, 125.3, 124.5, 123.6, 120.3, 20.5.

#### 3.2.3 Synthesis of 2-((5-(4-(di-p-tolylamino)phenyl)thiophen-2-yl)methylene)malononitrile (**1**)

To a solution of compound **4** (50 mg, 0.13 mmol) and malononitrile (13 mg, 0.19 mmol) dissolved in toluene (3 mL), and a drop of piperidine was added under a nitrogen atmosphere. The mixture was refluxed for 3 h. After cooling to room temperature, the solvent was evaporated by a rotary evaporator. The residue was purified by column chromatography (silica gel, ethyl acetate/petroleum ether = 1:10) to give the title compound **1** as a red solid (47 mg). Yield: 70%. ^1^H-NMR (CDCl_3_): δ 7.73 (s, 1H), 7.66 (d, *J* = 4.0 Hz, 1H), 7.49 (d, *J* = 8.8 Hz, 2H), 7.30 (d, *J* = 4.1 Hz, 1H), 7.13 (d, *J* = 8.2 Hz, 4H), 7.04 (d, *J* = 8.3 Hz, 4H), 6.98 (d, *J* = 8.7 Hz, 2H), 2.35 (s, 6H); ^13^C-NMR (CDCl_3_): δ 157.5, 150.3, 150.2, 143.9, 140.5, 134.2, 132.8, 130.2, 127.5, 125.6, 123.8, 122.9, 120.4, 114.6, 113.7, 74.5, 20.9; HRMS (ESI): *m*/*z*, [M + H]^+^ calcd. for C_28_H_22_N_3_S^+^, 432.1529; found, 432.1516.

### 3.3. General Fluorescence Spectra Measurements

Figure S4 shows that the fluorescence intensity of probe **1** (5 μM) was slightly affected within a range of pH (7.0–9.0) in the absence and presence of CN^−^ (50 μM) in PBS/DMSO (4/6) solution. The fluorescence responses of probe **1** were investigated in PBS/DMSO (4:6, *v*/*v*, pH = 7.4) solution. The response time of the probe **1** towards CN^−^ was 80 min (Figure S2). To ensure the reaction accomplish completely, fluorescence measurements were delayed for 1.5 h after anion addition.

Probe **1** was dissolved in DMSO to afford a concentration of 5 mM stock solution and then diluted to 5 μM with PBS/DMSO= 4/6 (*v*/*v*, pH = 7.4). Cyanide anion (10 mM) was offered by tetrabutylammonium cyanide. Moreover, miscellaneous interference anions (as their Na^+^ or K^+^ salt, 10 mM) including S^2−^, HS^−^, SO_3_^2−^, S_2_O_3_^2−^, S_2_O_8_^2−^, I^−^, Br^−^, Cl^−^, F^−^, NO_2_^−^, N_3_^−^, SO_4_^2−^, SCN^−^, HCO_3_^−^, CO_3_^2−^ and AcO^−^ in deionized water were used for the sensing experiments. The fluorescence spectrum changes caused by CN^−^ (50 μM) and miscellaneous interference anions (50 μM) were recorded at room temperature using a fluorescence spectrometer (λ_ex_ = 370 nm, λ_em_ = 480 nm, slits: 2.5 nm/5 nm).

### 3.4. Cell Culture and Confocal Fluorescence Imaging

HeLa cells were obtained from the State Key Laboratory of Biotherapy of Sichuan University. Cells were seeded to a 12-well plates, and were routinely maintained at 37 °C in a humidified 5% CO_2_ atmosphere using DMEM Growth medium with 10% fetal bovine serum for 24 h before use.

HeLa cells (Figure 6) were incubated with probe **1** (10 µM) for 1 h, and then washed with PBS five times. All cells were incubated with PBS for 15 min; finally with different concentrations of CN^−^ were added for 1 h at 37 °C: A, E with PBS; B, F with 10 µM CN^−^; C, G with 80 µM CN^−^; D, H with 160 µM CN^−^. Then cells were washed with PBS five times. Fluorescence images were monitored at 425−520 nm for blue channels and the white light channel.

Next, HeLa cells (Figure 7) were incubated with different concentrations of probe **1** for 1 h: A, E with PBS; B, F with 2.5 µM probe **1**); C, G with 5 µM probe **1**; D, H with 10 µM probe **1**. Then cells were washed with PBS five times. Then all cells were incubated with 80 µM CN^−^ for 15 min. Cell imaging was carried out after washing cells with PBS five times.

## 4. Conclusions

In summary, a colorimetric fluorescent probe **1** bearing triphenylamine-thiophene and dicyanovinyl groups has been synthesized. Probe **1** exhibited prominent selectivity and sensitivity for detecting cyanide anion with a significant turn-on fluorescent response in PBS/DMSO (4/6, *v*/*v*, pH = 7.4) solution. Moreover, a low detection limit (LOD, 51 nM) was achieved and it was noted that the probe **1** has potential for detecting level of cyanide anion in drinking water according to the requirements of the WHO (1.9 µM). In addition, good cell membrane permeability and low cytotoxicity to HeLa cells were also observed, suggesting its promising potential in bio-imaging.

## Supplementary Materials

The following are available online at www.mdpi.com/1424-8220/17/2/405/s1: Additional spectral data for probe **1** and spectroscopic characterization of compounds **1**–**3**.
