# Supplementary material for: A Biocompatible Colorimetric Triphenylamine- Dicyanovinyl Conjugated Fluorescent Probe for Selective and Sensitive Detection of Cyanide Ion in Aqueous Media and Living Cells
Source: Sensors (Basel). 2017 Feb 19;17(2):405. doi: 10.3390/s17020405 (PMC5336021; doi:10.3390/s17020405)
Supplement: Supplementary file 1 [file sensors-17-00405-s001.pdf]

Supplementary Materials: A Biocompatible Colorimetric Triphenylamine-Dicyanovinyl Conjugated Fluorescent Probe for Selective and Sensitive Detection of Cyanide Ion in Aqueous Media and Living Cells

Zi-Hua Zheng, Zhi-Ke Li, Lin-Jiang Song, Qi-Wei Wang, Qing-Fei Huang and Li Yang

Table of Contents

Figure S1.

Figure S2.

Figure S3.

Figure S4.

Figure S5. ^{1}H-NMR Spectrum of **3** in $CDCl_3$ (300 MHz).

Figure S6. ^{1}H-NMR Spectrum of **4** in DMSO-d_6 (300 MHz).

Figure S7. ^{13}C-NMR Spectrum of **4** in DMSO-d_6 (75 MHz).

Figure S8. ^{1}H-NMR Spectrum of **1** in $CDCl_3$ (300 MHz).

Figure S9. ^{13}C-NMR Spectrum of **1** in $CDCl_3$ (75 MHz).

Figure S10. HRMS (ESI) Spectrum of **1**.

Figure S11. HRMS (ESI) Spectrum of **[1 + CN]$^-$**

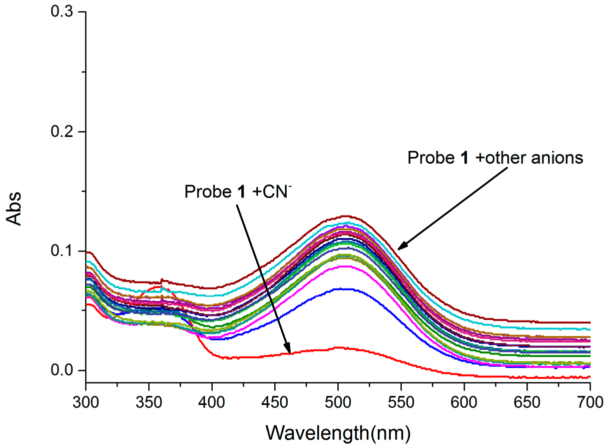

Figure S1. Changes in the absorption spectra of **1** (5 μM) upon addition of various anions (50 μM) in PBS/DMSO (4/6, pH = 7.4) solution.

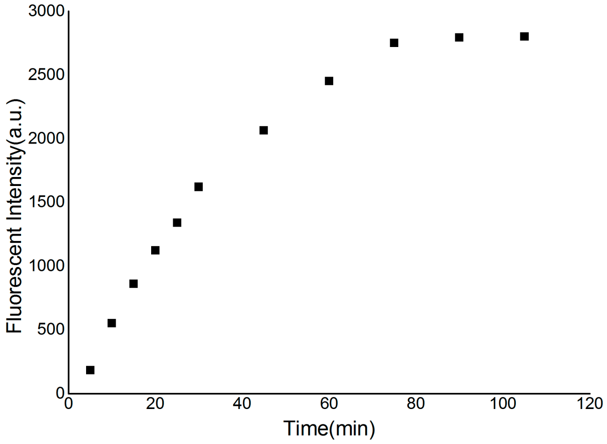

Figure S2. Time-dependent change of fluorescent intensity of probe **1** in absence or presence of CN^- (50 μM) in PBS/DSMO (4/6, pH = 7.4) solution. λ_{ex} = 370 nm, slit: 2.5 nm/5 nm.

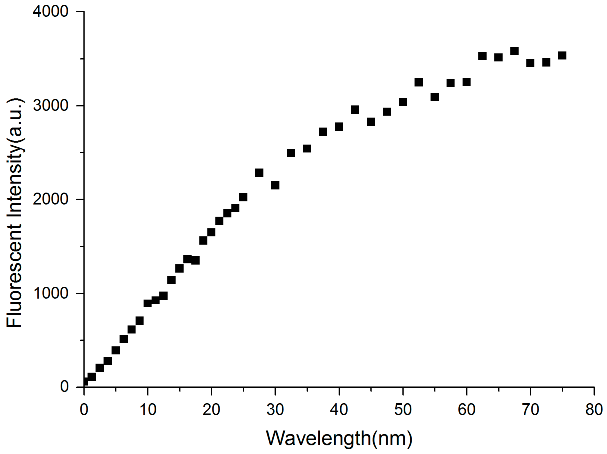

Figure S3. Fluorescent intensity of probe **1** (5 μM) at 480 nm upon addition of different concentration of CN^- (0–75 μM) in PBS/DSMO (4/6, pH = 7.4) solution. λ_{ex} = 370 nm, Slits: 2.5 nm/5 nm.

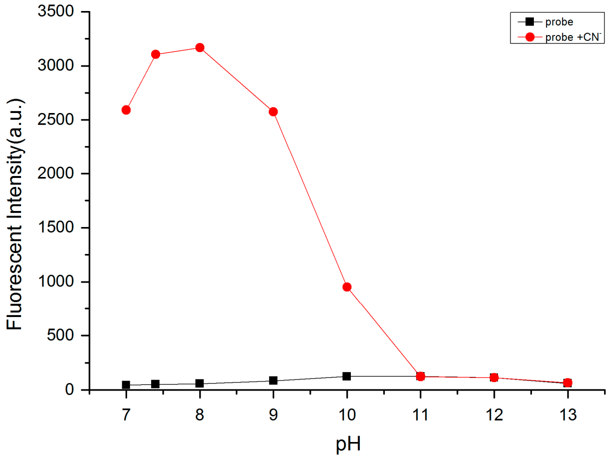

Figure S4. The effect of pH on the fluorescent intensity of probe **1** (5 μM) in the absence and presence of CN^- (50 μM) in PBS/DSMO (4/6) solution. Black line, probe; red line, probe + CN^-. λ_{ex} = 370 nm, Slit: 2.5 nm/5 nm.

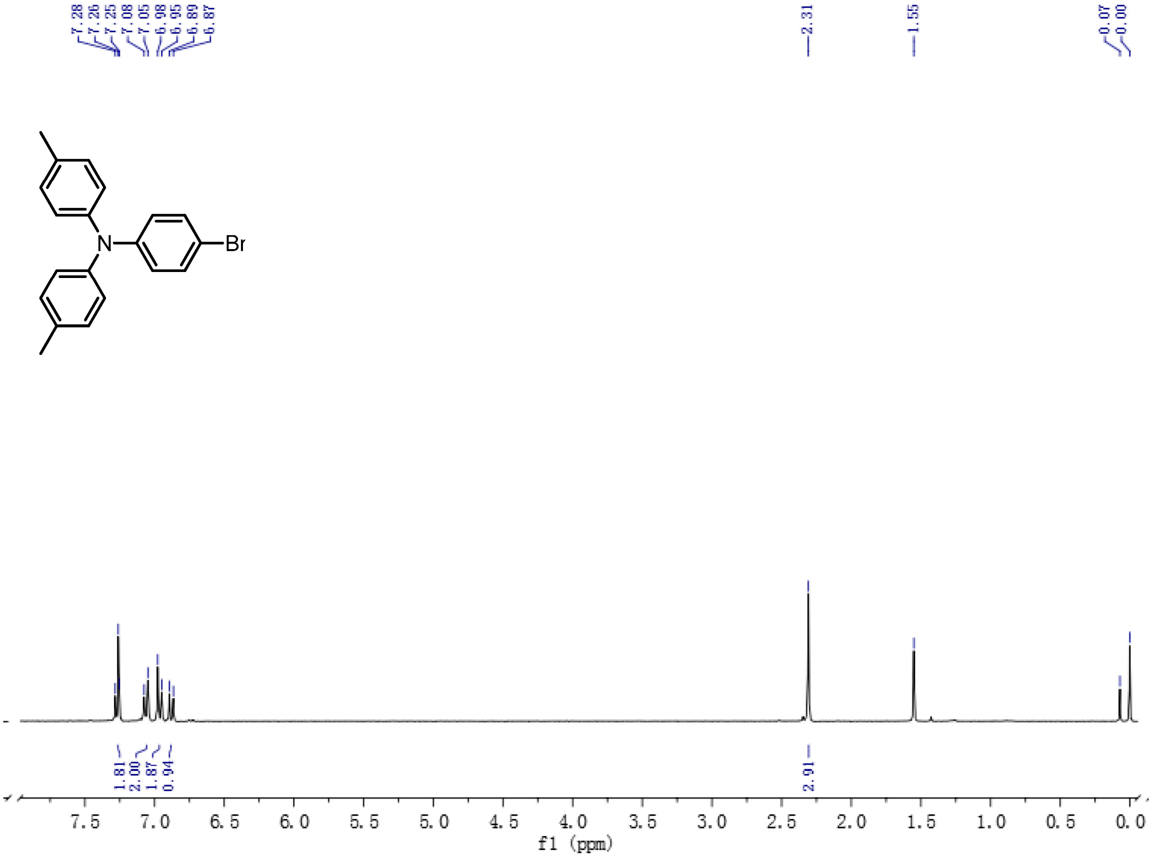

Figure 5. ^{1}H-NMR Spectrum of **3** in $CDCl_3$ (300 MHz).

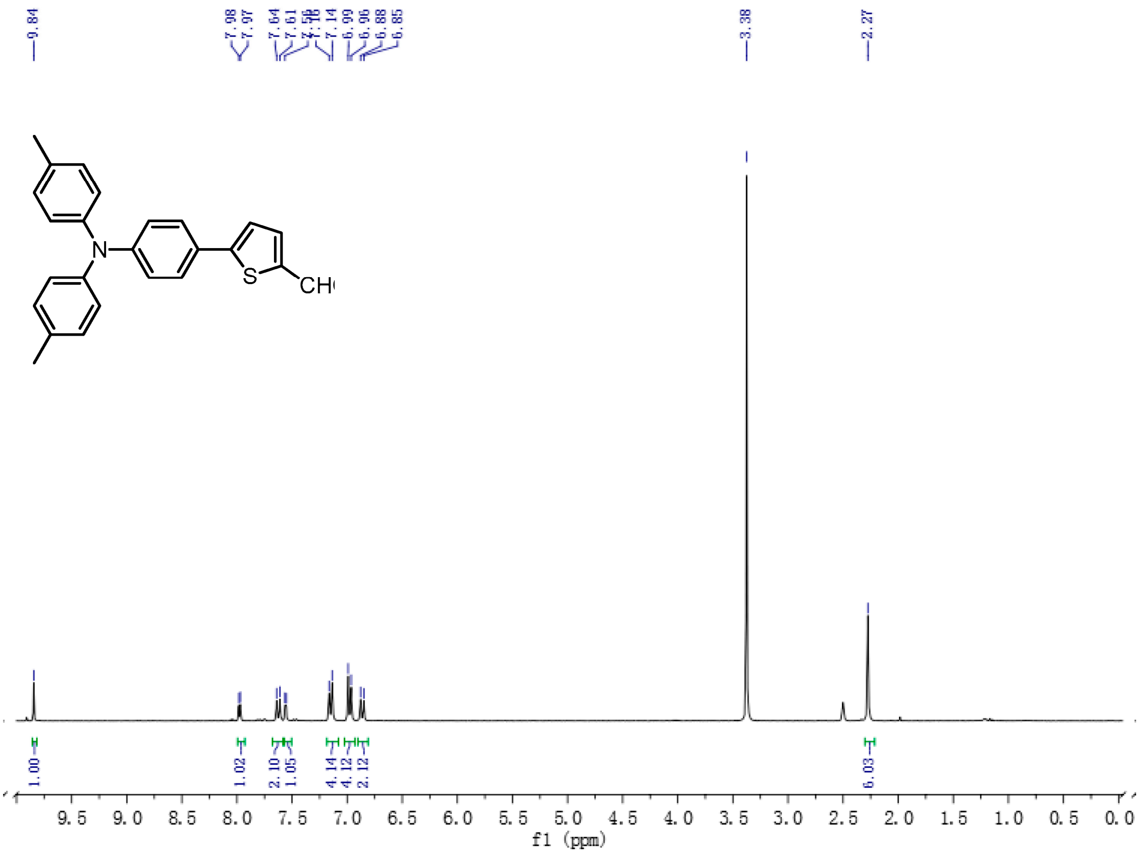

Figure S6. ^{1}H-NMR Spectrum of **4** in DMSO-d_6 (300 MHz).

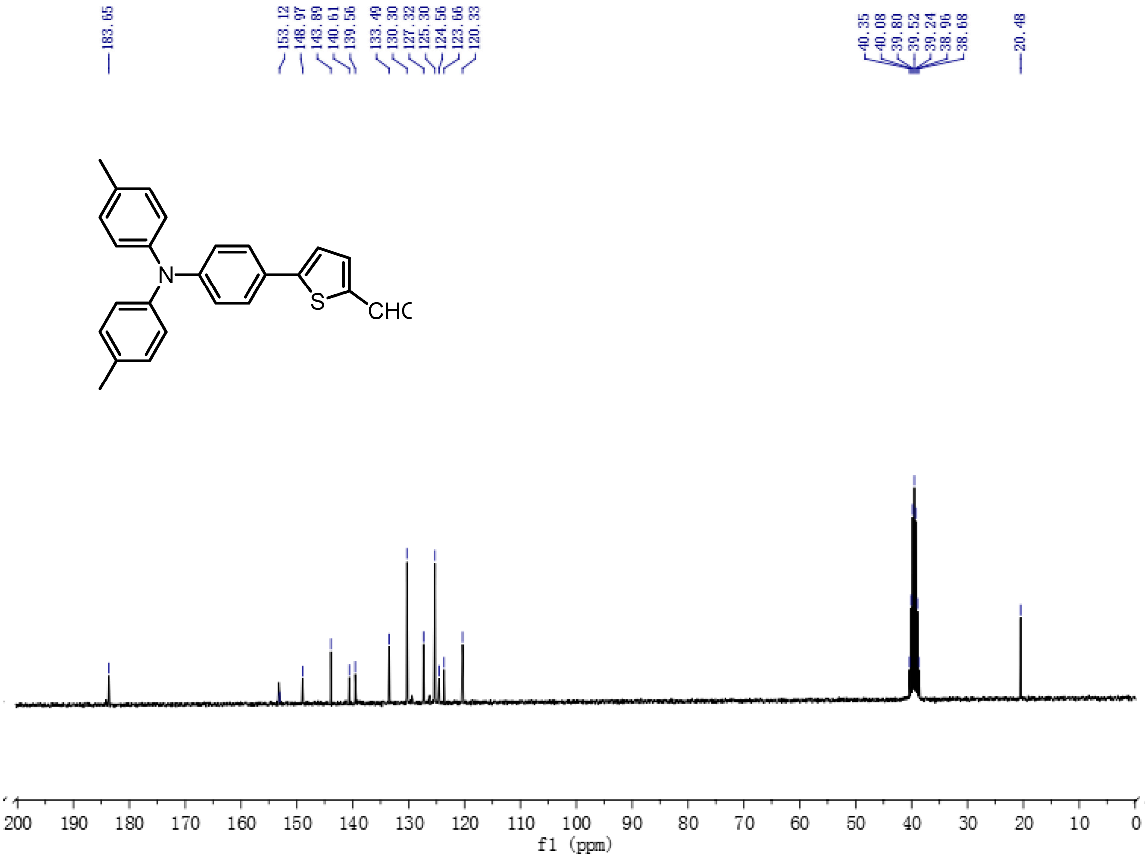

Figure S7. ^{13}C-NMR Spectrum of **4** in DMSO-d_6 (75 MHz).

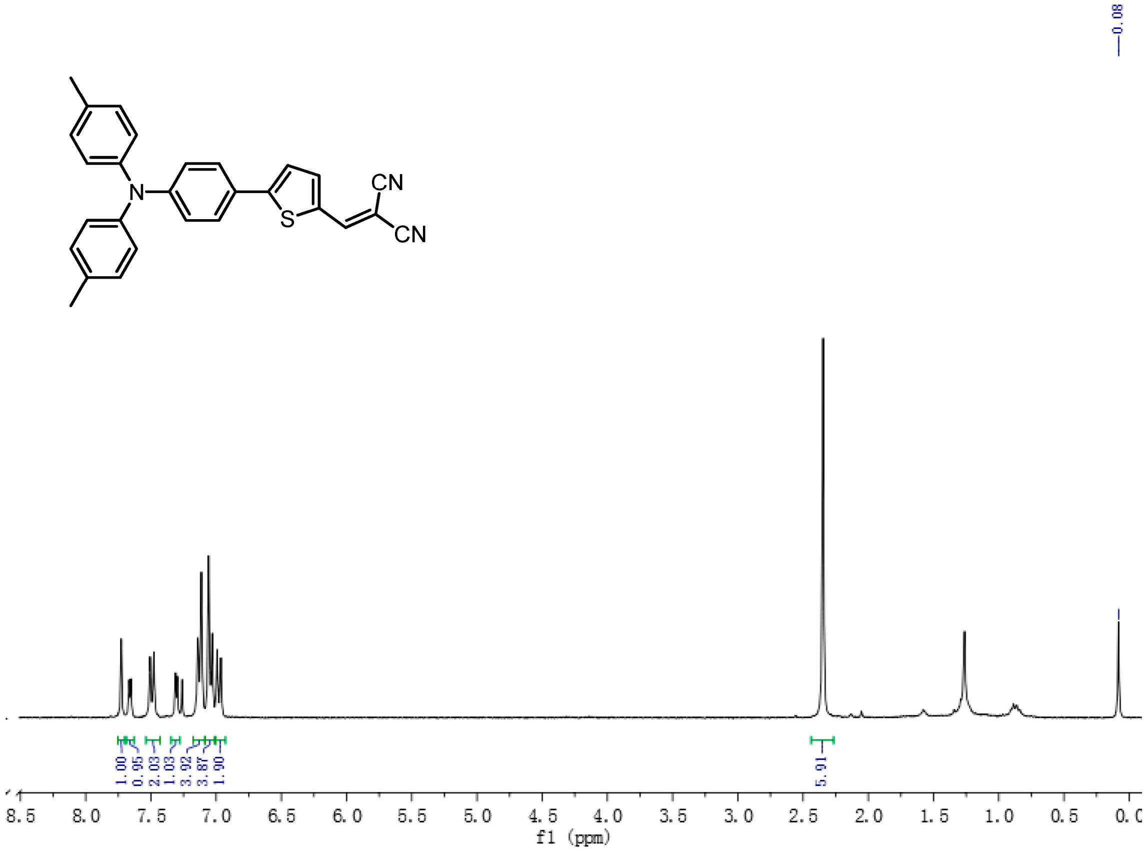

Figure S8. ^{1}H-NMR Spectrum of **1** in $CDCl_3$ (300 MHz).

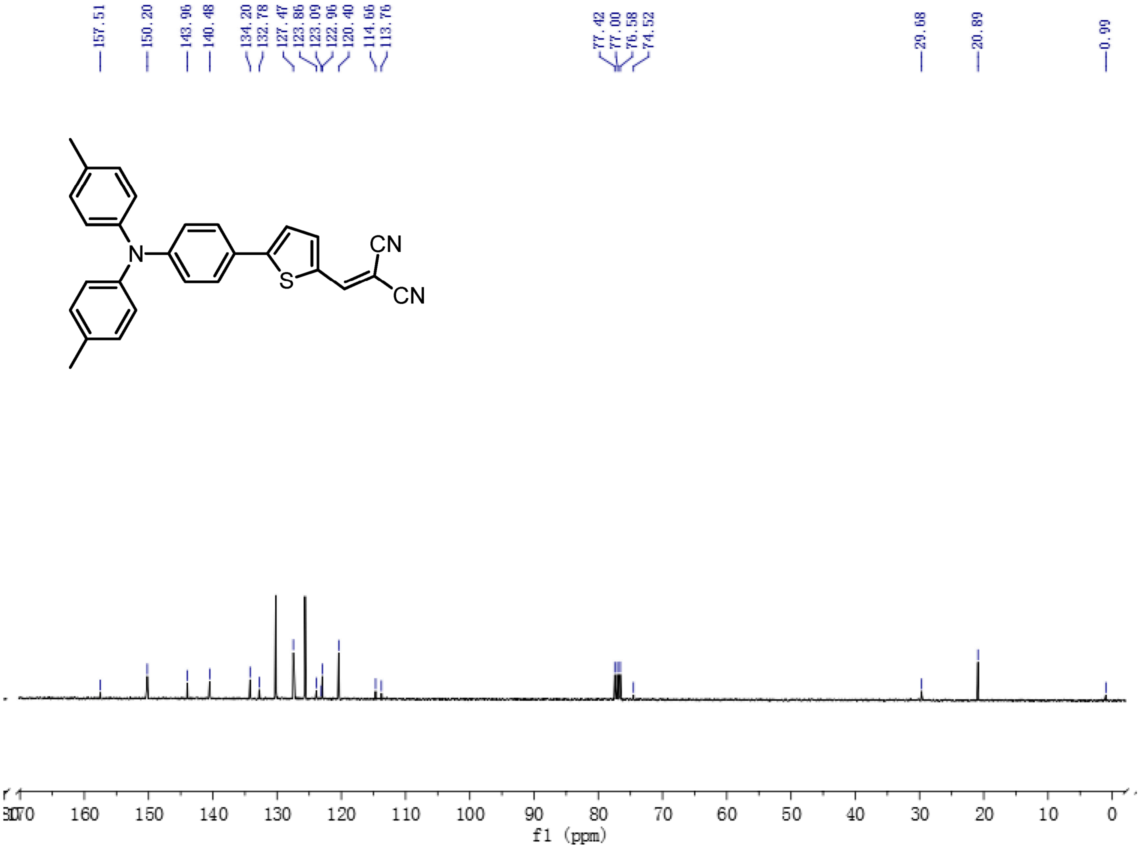

Figure S9. ^{13}C-NMR Spectrum of **1** in $CDCl_3$ (75 MHz).

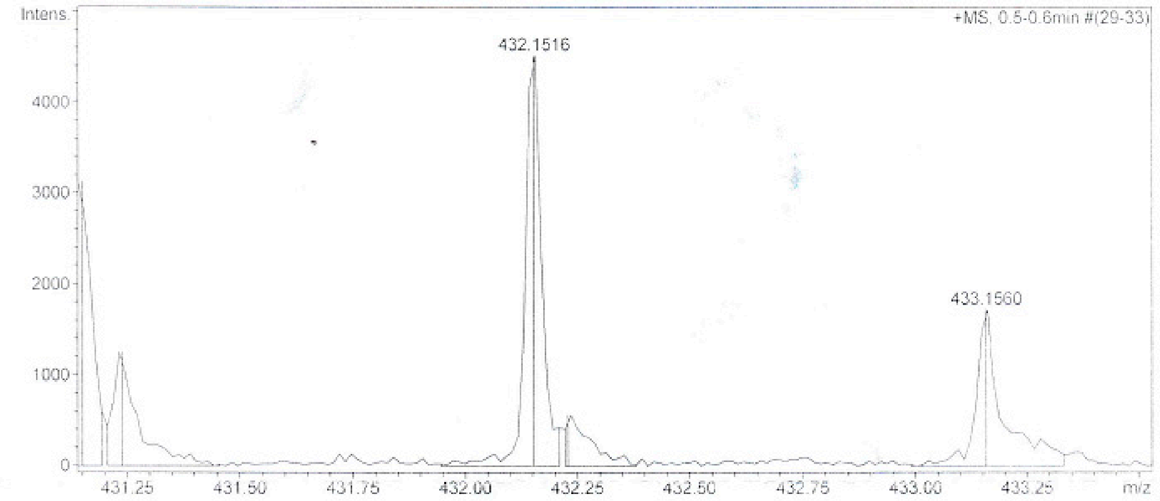

Figure S10. HRMS (ESI) Spectrum of **1**. calcd. for $[M + H]^+$ 432.1529, found 432.1516.

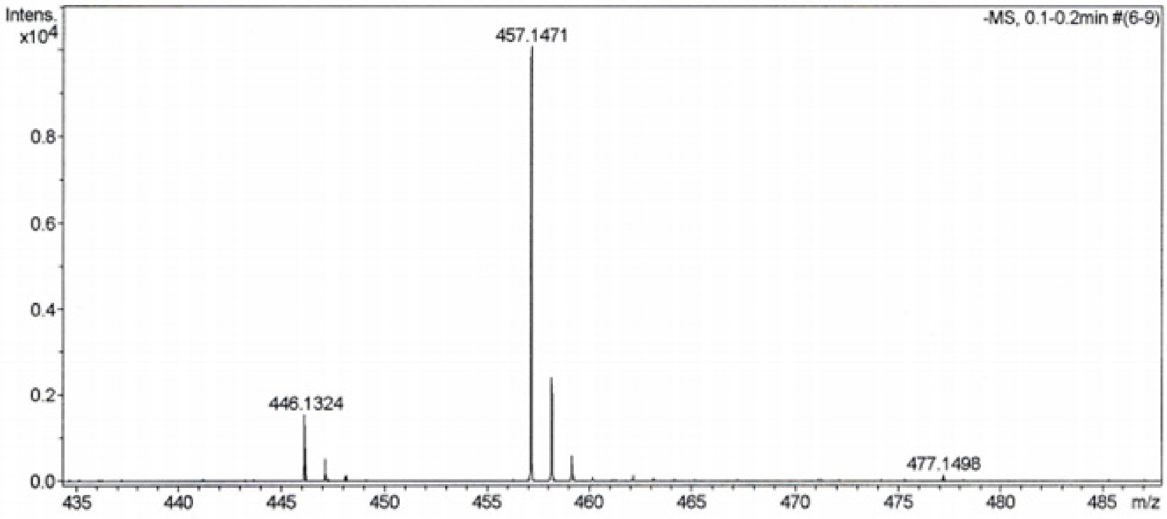

Figure S11. HRMS (ESI) Spectrum of **[1 + CN]⁻** calcd. for $[M]^-$ 457.1492, found 457.1471.
